# The TLR4 Agonist Immunomax Affects the Phenotype of Mouse Lung Macrophages during Respiratory Syncytial Virus Infection

**Published:** 2018

**Authors:** A. A. Nikonova, A. V. Pichugin, M. M. Chulkina, E. S. Lebedeva, A. R. Gaisina, I. P. Shilovskiy, R. I. Ataullakhanov, M. R. Khaitov, R. M. Khaitov

**Affiliations:** NRC Institute of Immunology FMBA of Russia, Kashirskoe shosse, 24, Moscow, 115478, Russia; Mechnikov Research Institute for Vaccines and Sera, Maliy Kazenniy Lane, 5A, Moscow, 105064, Russia

**Keywords:** bronchial asthma, macrophages, PRR, respiratory syncytial virus, TLR4 agonists

## Abstract

In the study, the effect of the TLR4 agonist Immunomax was investigated *in vitro *and *in vivo*. In particular, Immunomax was shown to polarize mouse bone marrow macrophages from the M0 and M2 states into the M1 state (*ARG1 *and *iNOS *mRNA expression levels were used to identify the mouse M1 and M2 phenotypes). Next, we investigated the prophylactic antiviral effect of Immunomax in both a model of mouse respiratory syncytial virus (RSV) infection and a model of RSV-induced bronchial asthma (BA) exacerbation. In the experiment with RSV-induced BA exacerbation, Immunomax-treated mice were characterized by a significant decrease of the viral load in lung homogenates, an increased amount of M1 macrophages in the lung, a tendency toward Th2-dependent ovalbumin-specific IgG1 antibodies decrease in blood serum, a significant increase in RSV-activated CD4+ T cells secreting IFNγ (Th1 cells), and a simultaneous significant decrease in the amount of CD4^+^ cells secreting IL-4 (Th2 cells) in the mouse spleen, which were detected by ELISPOT 1.5 months after experiment. These findings suggest that treatment with the TLR4 agonist Immunomax polarizes the immune response towards antiviral Th1 and may be used for short-term antiviral prophylaxis to prevent acute respiratory viral infections in asthmatics.

## INTRODUCTION

Bronchial astma (BA) is a disease associated with a chronic inflammation of the respiratory tract. Inflammation underlying bronchial hyperreactivity develops under the influence of type 2 T-helpers (Th2-response). Over the past 15–20 years, the prevalence of BA in the Russian Federation population has increased more than 3-fold and amounted to 902.8 per 100,000 (2007). In most cases, BA exacerbations in children and adults are associated with acute respiratory viral infections (ARVIs). Some viral species, such as the respiratory syncytial virus (RSV), rhinoviruses, metapneumovirus, influenza, parainfluenza viruses, and coronaviruses, are detected in the fluids of the respiratory tract during BA exacerbation [1]. In this regard, the development of new medical products of ARVI prevention is an important health care issue.

Alveolar Mφs (aMφs) are the most abundant cell population of bronchoalveolar lavage (BAL); their distinctive feature is the ability to acquire various phenotypes based on microenvironmental signals (classically activated M1, alternatively activated M2). The regulatory role of various Mφ phenotypes *in vivo *has been substantially studied; however, there is data indicating that Mφs may be involved in the pathogenesis of BA [2, 3].

Amongst pathogen pattern recognition receptors (PRRs), the most studied are Toll-like receptors (TLRs), activation of which is necessary for triggering mechanisms of the innate immune response to infection. For this reason, TLR agonists are believed to be potential immunotherapeutic agents or vaccine adjuvants for the treatment of infectious diseases. Immunomax, a TLR4-agonist isolated from potato sprouts [5], is effective against a number of viral (papillomavirus, herpes virus) and bacterial pathogens and likewise exhibits potential antitumor activity [6]. Earlier, the immunomodulator Immunomax was reported to activate monocytes, Mφs, NKs, and dendritic cells [7].

In this study, we investigated the role of M2 Mφs in BA exacerbation induced by viral infections, as well as the possibility of influencing the Mφ polarization from M2 to M1 using TLR agonists in order to increase the effectiveness of immune defense against RSV under an allergic immune response.

## EXPERIMENTAL

**Polarization of mouse macrophages by Immunomax *in vitro***

**Fig. 1:**
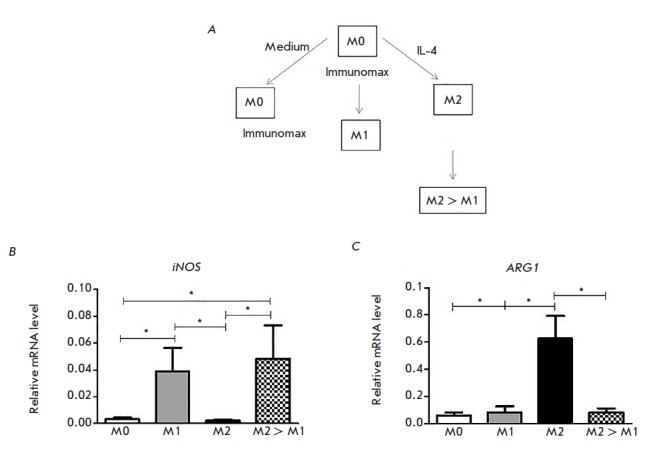
A TLR4 agonist Immunomax polarizes *in vitro *mouse macrophages towards the M1 phenotype. (*A*) Design of the experiment; *iNOS *(*B*) and *ARG1 *(*C*) mRNA expression measured by RT-PCR

Macrophages (Mφs) derived from mouse bone marrow were placed in 24-well plates at a concentration of 105 cells per well and cultured in a medium containing the granulocyte-macrophage colony-stimulating factor (GM-CSF) for 7 days. After the 7 days, the cells were treated for 24 hour with IL-4 to obtain M2 Mφs, Immunomax to obtain M1, or the medium alone to produce M0. Twenty-four hours after treatment with IL-4, the medium was replaced with a new one containing Immunomax for repolarization of M2 into M1; in addition, Immunomax was added to M0 for repolarization to M1. The experiment scheme is shown in *Fig. 1A*. Twenty-four hours after treatment, cell lysates were collected, frozen at –80 °C, and stored until the analysis. Next, the samples were analyzed by RT-PCR to determine mouse *iNOS* and the *ARG1 *mRNA levels.

***In vivo *experiments**

**Fig. 2:**
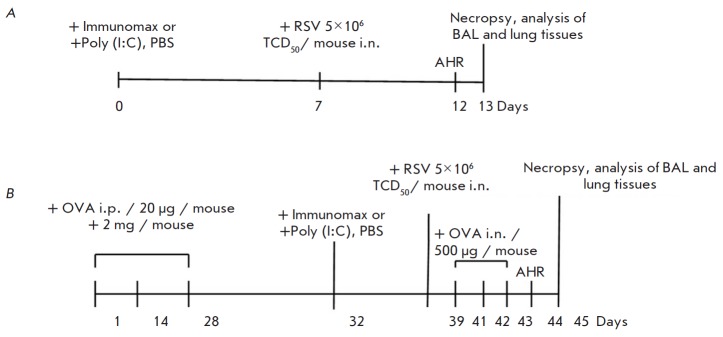
Experimental protocol of animal sensitization, challenge, RSV infection, and TLR agonist treatment. (*A*) The mouse model of RSV infection; (*B*) the mouse model of RSV-induced BA exacerbation. PBS – phosphate-buffered saline, i.n. – intranasal introduction, AHR – airway hyperresponsiveness, OVA – ovalbumin, i.p – intraperitoneal injection, BAL – bronchoalveolar lavage. The following experimental animal groups were used (RSV infection model): (1) RSV+Immunomax (*N*=17); (2) RSV+Poly (I:C) (*N*=17); (3) RSV (*N*=17); (4) intact mice (*N*=17). The RSV-induced BA exacerbation model: (1) BA/RSV+Immunomax (*N*=17); (2) BA/RSV+Poly (I:C) (*N*=17); (3) BA/RSV (*N*=17); (4) intact mice (*N*=17)

We assessed the prophylactic effect of Immunomax *in vivo *in two experimental models: RSV infection in mice and virus-induced BA exacerbation in mice. The experiment schemes are presented in *Fig. 2A,B*, respectively. The RSV strain A2 was chosen as a viral agent. Female BALB/c mice weighing 18–20 g received from Stolbovaya (Russia) were used in the experiments.

For the experiments, four groups of animals were formed (*N *= 17) (*Fig. 2*). Eight mice in each group were used for histological examination and PCR analysis. A number of mice (*N *= 4) were used to analyze the phenotypes of lung macrophage populations (by means of complex cell analysis of bronchial lavage and multiparameter flow cytometry of lung cell infiltrate). Four mice from each group were used to determine the amount of RSV-specific CD4^+^ T cells secreting IFN-γ and IL-4 by the ELISPOT method 1.5 months after infection.

Based on the study by Misharin *et al*. [8], we selected the following combination of antibodies to identify myeloid cell populations: CD11b Brilliant Violet 510™, CD45 AlexaFluor®, Ly-6C PE, CD11cPE/Dazzle™ 594, CD49bPerCP/Cy5.5, Ly-6GPE/Cy7, F4/80APC, and IA/I-EAPC/Cy7. FACSAria II flow cytometer was used to sort cells. The expression of *iNOS *and *ARG1 *mRNAs in sorted Mφs was evaluated by RT-PCR.

To evaluate the antiviral effect of TLR agonists, the RSV RNA level in the lysates of cells isolated from lung homogenates was determine by RT-PCR.

RSV-specific CD4^+^ T cells secreting IFN-γ and IL-4 were identified using the commercial Mouse IL-4 ELISPOT Set and Mouse IFN-γ ELISPOT Kit (BD Biosciences, USA), according to the manufacturers’ instructions.

The statistical analysis was performed with the GraphPad Prism version 4.0 software. Data was considered statistically significant at P < 0.05.

## RESULTS AND DISCUSSION

We studied the ability of Immunomax, a TLR4 agonist, to polarize mouse macrophages from M0 and M2 into M1. The Mφ phenotype was identified based on the expression of mRNA of the *iNOS *and *ARG1 *genes – markers of the M1 and M2 phenotypes, respectively [9]. Our findings confirm the ability of Immunomax to repolarize cells to the M1 state. In particular, Immunomax-treated M0s were characterized by increased *iNOS *expression and reduced *ARG1 *expression. We observed the same effect in Immunomax-treated M2 cells (an increase in the *iNOS *mRNA level and a decrease in the *ARG1 *mRNA level (*Fig. 1B,C*)).

At the next stage, we studied *in vivo *the prophylactic effect of Immunomax in both experiments of mouse RSV infection and experiments of RSV-induced BA exacerbation (*Fig. 2*). In these experiments, we evaluated a number of parameters, such as the lung function, BAL cell composition, histological alteration, and level of serum ovalbumin-specific antibodies of different classes (IgE, IgG1, and IgG2). There were no statistically significant differences in the values of these parameters in the animals of the experimental groups (data not shown).

However, it should be noted that in the experiment with BA + RSV, we established a tendency of the ovalbumin-specific IgG1 antibody level in the serum of Immunomax-treated mice to decrease (data not shown). Th2-dependent IgG1 antibodies are classic carriers of antibody properties, the level of which may increase in allergic diseases. A decrease in the level of IgG1 antibodies under the influence of Immunomax, which was observed in this study, seems to indicate the development of Th1-type immune responses. Interestingly, these responses were observed at the systemic level, despite intranasal administration of the agent.

**Fig. 3:**
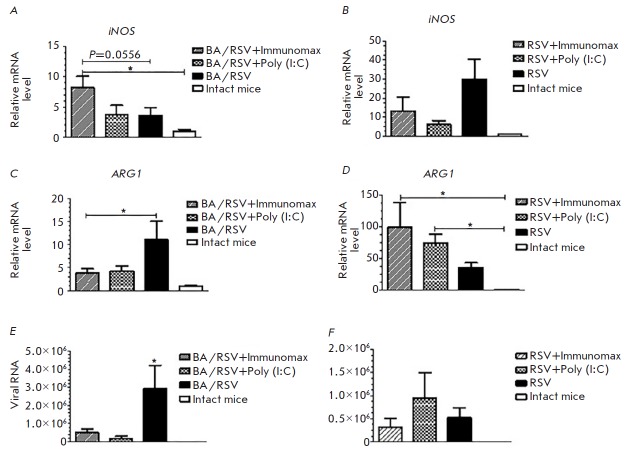
*iNOS *(*A, B*) and *ARG1 *(*C, D*) mRNA expression in mouse alveolar macrophages and (E, F) viral load in lung tissue (RSV RNA copies per 1 g of lung tissue) measured by RT-PCR. (*A*, *C*, *E*) the RSV-induced BA exacerbation model; (*B*, *D*, *F*) the mouse model of RSV infection

Of particular interest was the phenotype of mouse pulmonary Mφs in different experimental conditions, because we supposed that intranasally administered Immunomax would act on these cells, polarizing them into the M1 state. To test this hypothesis, we isolated Mφs from the lungs by sorting cells with a FACSAria II flow cytometer and evaluated the expression of *iNOS *and *ARG1 *mRNAs. An analysis of the RT-PCR data revealed an increase in the *iNOS *level (*Fig. 3A*) and a significant decrease in the *ARG1 *level (*Fig. 3C*) in Mφs of Immunomax-treated mice with RSV- induced BA exacerbation. This indicates polarization of Mφs into the antiviral M1 state. However, in the mouse RSV infection experiment, this tendency was not revealed (*Fig. 3B,D*).

Assessment of the viral load revealed a statistically significant decrease in the viral RNA level in the lungs of Immunomax-treated mice compared to that in untreated animals (*Fig. 3E*). There was also a reduction in the viral load in a group treated with a TLR3 agonist, Poly (I:C). These data were obtained in the experiment with virus-induced BA exacerbation. We also found a decrease in the viral load in RSV-infected mice treated with the studied agent (*Fig. 3F*).

**Fig. 4:**
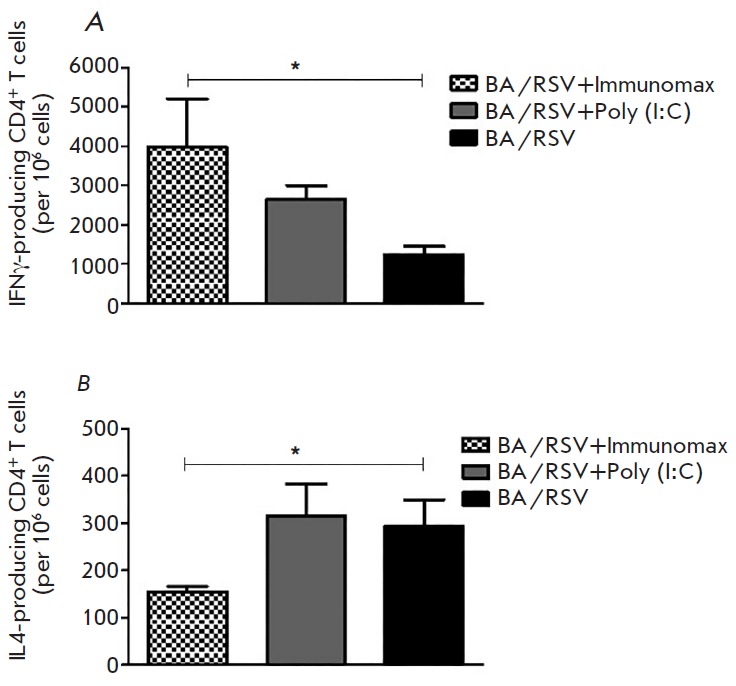
Amount of IFNγ (A) and IL-4 (B) producing CD4+ T cells in the spleen of mice with RSV-induced BA exacerbation (mice were maintained up to 1.5 months after the experiment), determined by ELISPOT (cells were stimulated by UV-inactivated RSV)

*Figure 4* presents the results of identification of RSV-specific Th1 and Th2 cell differentiation using the ELISPOT method. In the experiment with the model of RSV-induced BA exacerbations, we observed a significant increase in RSV-activated CD4+ T cells secreting IFN-γ (Th1 cells) (*Fig. 4A*) and simultaneously a significant decrease in CD4+ cells secreting IL-4 (Th2 cells) (*Fig. 4B*) in the spleen of Immunomax-treated mice. In Immunomax-treated mice with RSV infection, there was an increase in the level of activated CD4+ T cells secreting IFN-γ (Th1 cells), while no CD4+ cells secreting IL-4 (Th2 cells) were detected (data not shown).

Therefore, *in vitro *and *in vivo *experiments demonstrated that the TLR4 agonist Immunomax is able to re-polarize the Th2 response to the antiviral Th1 state. The effect of Immunomax was observed on the model of RSV-induced BA exacerbation where a pronounced Th2 response was initially induced in mice by sensitization with a model allergen ovalbumin. The basal concept in the treatment of allergic diseases is an inversion of the immune Th2 response towards Th1. This approach is especially important for BA patients, because abundant data has indicated a more severe course of ARVIs in asthmatics [9]. This is believed to be related to the dominant Th2 response that is probably associated with the prevalence of activated M2 Mφs in the lungs of patients [3]. In this regard, our findings indicate that treatment with Immunomax, a TLR4 agonist, affects the phenotype of pulmonary Mφs, polarizing the immune response towards Th1, and, therefore, that it may be used for the prophylaxis of RSV infection in asthmatics.

## Abbreviations

BA: bronchial asthma
ARVI: acute respiratory viral infection
IL: interleukin
RSV: respiratory syncytial virus
TLR: Toll-like receptor
Mφ: macrophage
IFN: interferon
i.n.: intranasal administration
i.p.: intraperitoneal administration
BAL: bronchoalveolar lavage
RT-PCR: real-time PCR
aMP: alveolar MP

## References

[1] Tsarev S.V., Khaitov M.R. (2009). Rus Med J..

[2] Nikonova A.A., Khaitov M.R., Khaitov R. M. (2017). Medical immunology..

[3] Melgert B.N., ten Hacken N.H., Rutgers B., Timens W., Postma D.S., Hylkema M.N. (2011). J. Allergy Clin. Immunol..

[4] Ataullakhanov R.I., Pichugin A.V., Melnikova T. M., Khaitov R.M. (2013). Patent, RU2013151824A, Russia, A61P 37/02,.

[5] Ghochikyan A., Pichugin A., Bagaev A., Davtyan A., Hovakimyan A., Tukhvatulin A., Davtyan H., Shcheblyakov D., Logunov D., Chulkina M. (2014). J. Transl. Med..

[6] Bagaev A., Pichugin A., Nelson E.L., Agadjanyan M.G., Ghochikyan A., Ataullakhanov R.I. (2018). J. Immunol..

[7] Misharin A.V., Morales-Nebreda L., Mutlu G.M., Budinger G.R., Perlman H. (2013). Am. J. Respir. Cell Mol. Biol..

[8] Murray P.J., Wynn T.A. (2011). J. Leukoc. Biol..

[8] Corne J.M., Marshall C., Smith S., Schreiber J., Sanderson G., Holgate S.T., Johnston S.L. (2002). Lancet..

